# Position to Exchange

**Published:** 1895-08

**Authors:** 


### POSITION TO EXCHANGE.

A TWELVE-HUNDRED-DOLLAR a year army position, carrying with it allowances of quarters, light, and fuel, with privilege of outside practice, for an equally remunerative position or practice in or near a city.

A pleasant station, delightful climate, especially beneficial to invalids; splendid hunting and fishing. Address "Exchange," care of The Journal of Comparative Medicine and Veterinary Archives, 3452 Ludlow Street, Philadelphia, Pa.

